# A Case of a Strangulated Hernia Successfully Treated; to Which Are Added Some Remarks on the Use of Cold Applications in the Reduction of Herniæ

**Published:** 1785

**Authors:** 


IV. *A Caſe of a ſtrangulated Hernia ſucceſsfully treated; to which are added ſome Remarks on the Uſe of cold Applications in the Reduction of Herniæ. Communicated in two Letters to Dr.* Simmons, *by Mr.* William Cribb, *Surgeon at Biſhop's Stortford, in Hertfordſhire.*

THOMAS Stiles, aged about ſixty years, coachman to a clergyman in this neighbourhood, was taken with a ſevere vomiting on Tueſday, November 16, 1784, which continued till Friday the 19th, when I firſt ſaw him.

him. He did not complain of much pain in any part, but ſaid his belly was tender, and aſſured me that his rupture was not down, and that he had worn his truſs all the time he had been ill. As what he brought up both looked and ſmelt like fæces, I ſuſpected that a hernia, by obſtructing the paſſage through the gut, might occaſion his preſent ſymptoms, and, on taking off the truſs, found a protruſion in the left groin about the ſize of an egg, but ſomewhat flatter. He had one ſtool the day after he was taken, which was probably only fæces below the ſtrangulation. His vomiting always came on with a hiccup; he had little or no tenſion of the abdomen, but the ſlighteſt preſſure in attempting to return the contents of the hernial ſac gave him great pain.

I took away ten ounces of blood; directed a gently ſtimulating clyſter to be given as ſoon as it could be prepared; and that of an ounce of Glauber's ſalt, diſſolved in a pint of water, he ſhould take four table ſpoonfuls every two hours till he had taken the whole; at the ſame time I ordered him to have large cloths wrung out of the coldeſt ſpring water, and applied every two or three minutes to the part till I ſhould ſee him again, which I promiſed to do in in ſix hours; but in about two hours after I left him it went up.

This ſtrangulation was probably induced by a tight truſs being put on, and continued to be worn when the hernia was down.

This is a caſe, among many others, in which I have experienced the benefit of cold applications in the reduction of herniæ. It is certain that whatever may be the remote cauſe of hernia, the proximate and evident one is a ſufficient enlargement of the abdominal ring or ſome other part, to ſuffer part of the contents of the abdomen to protrude, and which, owing to ſeveral concurring cauſes, cannot be returned; the principal of which is, firſt, either a ſtricture made by the tendinous fibres of the abdominal muſcles, or the part through which the protruſion appears, from ſome diſeaſed alteration in itſelf; or, ſecondly, from ſome alteration in the protruded parts.

The firſt of theſe cauſes, though maintained by ſeveral modern authors of deſerved reputation in their profeſſion, I believe, much leſs frequently occurs than is generally imagined, as in neither of thoſe caſes in which I have performed performed the operation for the bubonocele, nor in others, where I have had opportunities of inſpecting the bodies after death, were there any alteration in the natural appearance of the tendinous apertures. But the moſt common cauſe of ſtrangulated hernia ariſes from an alteration taking place in the parts contained in the hernial ſac ſubſequent to the protruſion; or, as an ingenious writer* well expreſſes it, "the ſtrangulation does not take place, be- "cauſe the opening is leſs than it was before "the ſymptoms came on, but becauſe the "parts ſo diſplaced are become larger." Suppoſe ſome of the contents of the belly to deſcend, it is neglected to be put up, and, from an unnatural tightneſs round a part of it, the blood is prevented from returning with the freedom it ought to do; this occaſions a turgidneſs and fulneſs of the vaſcular ſyſtem. If the hernia be inteſtinal, it probably contains fæces, from which the chyliferous veſſels are ſeparating the finer parts as long as they can do their office, and, when prevented, they alſo grow full and turgid, and the fæces become a little more ſolid than when firſt protruded; and it is alſo probable that more of the natural

* Wilmer's Caſes and Remarks, page 3.

fæcal fæcal contents of the inteſtines might be forced in from above after the parts are irreturnable: add to this that the air contained in the gut from the increaſed heat in conſequence of incipient inflammation, from general fulneſs and partial obſtruction, may be ſomewhat more rarified; all which tend to increaſe the bulk of the protruded parts. How then are warm applications, as the hot bath, fomentations, &c. to decreaſe this fulneſs? I am afraid they too frequently have a contrary effect; and what Mr. Bell, in his very uſeful ſyſtem of ſurgery, lately publiſhed, obſerves with reſpect to fomentations, poultices, and other means of applying local heat, may, in my opinion, be applied with equal propriety to the general warm bath, he ſays, "On the conſtricted tendon they can "have no influence, for it always lies ſo deep "as to be out of the way of every local appli-"cation of this nature; and as the heat con-"veyed by ſuch remedies muſt for certain "tend to rarify the contents of ſuch ſwellings, "by their thus producing an increaſe of ſize "in the tumors to which they are applied, in-"ſtead of anſwering any good purpoſe, on this "principle it is evident they muſt do harm*;"

* Vol. I. page 120.

they quicken the circulation, increafe, by their warmth, the expanfion of the parts, and, at the fame time, can have no effect, as is commonly fuppofed, of relaxing the abdominal ring; and I believe where the warm bath, fomentations, &c. have been fuppofed to be efficacious, it has been more owing to bleeding, clyfters, or other caufes, than to that to which it has been attributed. On the contrary, thofe methods which can be fuppofed in any way to leffen the bulk of the parts, or to abate inflammation, are the moft likely to do good. We know that cold poffeffes the property of condenfing and leffening the bulk of fluids, and is likewife the beft anti-phlogiftic application that we know. After the neceffary evacuations, and gentle attempts in the moft relaxed pofition of the abdominal mufcles on the difeafed fide, I generally direct large cloths, dipped in the coldeft water at hand, to be applied over the part, abdomen and fcrotum, and renewed every two or three minutes, and the water in the veffel (particularly in warm weather) to be changed every half hour. By thefe means a pretty confiderable degree of cold is induced on the part and patient, and the rupture commonly goes up with the patient's tient's own endeavours. In the cafe above ftated it went up in the courfe of a few hours; but I have known it neceffary to continue the procefs for fix, twelve, and even twenty-four hours, and fometimes longer. The cold thus produced generally abates the pain in the part, and has confiderable effect on the inflammatory fymptoms, as it decreafes the action of the vafcular fyftem both partially and generally; and it has this great recommendation in preference to warm applications, that the patient is not made worfe by it, which is moft univerfally the cafe after the contrary method has been purfued; and thofe who have had opportunities of feeing many of thefe cafes, have lamented the rapid increafe of formidable fymptoms after the patient has been once or twice in the warm bath, and the parts kept conftantly fomented with hot ftupes, &c.

It is no uncommon thing for the hernia to go up of itfelf, and fometimes with a kind of fhivering produced by the application of cold. In T. Stiles's cafe a greater degree of inflammation than is common in old herniæ was brought on by the preffure of the trufs, yet it was readily reduced after a fhort application of wet cloths.

In warm weather it is by ſome recommended to make the water artificially colder than in its natural ſtate. This I have never done but in two caſes: in one, a man about forty, I directed ice to be uſed in a diſſolving ſtate; but I had reaſon to believe it was partially and improperly applied, and I was under the neceſſity of relieving him by the operation, and he did well. The other, a woman of ſixty years of age, in which water, with crude ſal ammoniac, was uſed, and frequently renewed for two days, and at the end of the third, after every other method, both cold and hot, had been recommended and tried, ſhe was adviſed, from the urgency of the ſymptoms, to have it put up by an operation, which ſhe ſubmitted to; and it was remarkable that the wound, though five inches in extent, healed in the firſt or adheſive ſtage of inflammation, and was perfectly cicatriſed in a fortnight, and ſhe was enabled from that time to wear a ſteel truſs.

As I think it the duty of every practitioner to communicate his obſervations, ſo far as they relate to the treatment of diſeaſes, when there is any deviation, either in theory or practice, from the common or received opinion, I hope I ſhall be excuſed in offering theſe remarks; for although the practice now recommended, of applying cold in cafes of ftrangulated hernia, is not at prefent new*, yet I will venture to affert that it is not *generally* known, and, as far as my knowledge of the practice of others has extended, is very little ufed.

*Bifhop's Stortford, Herts,*
June 3, 1785.

---

This patient was again attacked, June 11th, with fymptoms of ftrangulated hernia. The next morning, when I faw him, he had a fmall tumor on the left fide, which fcarcely elevated the fkin; it was attended with conftant vomiting of excrementitious matter, and a very troublefome hiccough. The hernia was painful on the flighteft touch, and the belly tenfe; his pulfe hard and irregular: he was freely bled, and a clyfter of water gruel directed to be given every fix hours, and alfo a weak folution of Glauber's falt to be taken every four hours, and cloths dipped in cold water to be applied every two or three minutes. The 13th he was much the fame; the clyfters had brought

* See Monro, Edinb. Med. Effays, Bell, Wilmer, &c.

away a ſmall quantity of fæces; but he had diſcontinued the cold water in about an hour and a half after I left him. I preſſed him ſtrongly to a more conſtant and ſteady uſe of it; ordered the clyſters to be continued every ſix or eight hours, and repeated the bleeding. In the evening the inflammation was much abated, and the tumor was ſofter, leſs tenſe, and free from pain, as was alſo the abdomen.

The 14th the ſtrangulation was ſtill the ſame, the vomiting leſs violent, but the hiccough very ſevere: he was again bled, the wet cloths were continued, and he had a clyſter of the ſmoke of tobacco. In the evening Dr. R. Dimſdale, and Mr. Scarr, ſurgeon, both of this place, were ſo obliging as to viſit the patient with me. The man was eaſy and cool, but the pulſe was quick and irregular, with frequent retchings and hiccough: it was agreed to defer the operation till the next morning, and, in the mean time, he was ordered to take a weak ſolution of neutral ſalts.

On the 15th he was extremely reſtleſs; ſhewed great anxiety in his countenance; his pulſe was low, weak, and irregularly intermitting; his vomiting almoſt continual; his hiccough inceſſant; and his fleſh felt cold and dewy.

The

The operation was now become inevitable, and therefore I performed it in the preſence of the two gentlemen above mentioned.

On opening the peritoneal ſac, and expoſing the hernia, we found it to be a portion of large inteſtine, juſt enough to take in its whole diameter, and without any omentum. The abdominal ring, inſtead of occaſioning the ſtrangulation, was preternaturally enlarged. On a farther examination I diſcovered that the ſtricture was formed by the neck of the hernial ſac itſelf* more than an inch within the natural abdominal aperture, and the ſtricture was ſo tight, that it was not without great difficulty that I could introduce a ſmall probe director between the ſtricture and the gut, without the latter's collapſing over it. This was cut through, and the operation finiſhed in the common way; only, as the ſac was very much thickened and indurated, I thought it prudent to cut away the greater portion of what appeared externally, to prevent the cure from being retarded by its being left to ſlough away of itſelf. The cure was completed in

* See two caſes of this kind, mentioned by Mr. Wilmer in his Caſes, page 12.

twenty twenty days, ſince which time he has conſtantly worn his truſs.

I would here obſerve, that, from the extreme tightneſs of the ſtricture, which in this caſe occaſioned the ſtrangulation, it is evident that no external application could poſſibly have ſufficiently leſſened the protruded parts, ſo as to admit of their reduction; and it would be equally nugatory to talk of relaxing the thickened, indurated, and inelaſtic hernial ſac by any external means we are at preſent acquainted with.

In this caſe we may obſerve the good effects of cold in abating inflammation; for, after it had been ptoperly applied for a few hours, the pain and tenderneſs, both in the part itſelf and the belly, were almoſt totally removed, and did not afterwards return. I muſt acknowledge that I have been ſo fully convinced from this, and a variety of other caſes, of the ſuperiority of the cold to the warm method of treating this complaint, that I truſt to it entirely in all caſes of this kind, being ſatisfied that if we cannot ſucceed by condenſing and leſſening the protruded contents of the hernial ſac, we ſhall never ſucceed by expanding and enlarging them. I would only add, that as far as my obſervation goes, the parts under this treatment are in a better ſtate for the operation, if it becomes neceſſary, and the ſymptoms during the cure are more favourable than when treated in the oppoſite manner.

*Biſhop's Stortford,*

Auguſt 30, 1785.

